# Every Man His Own Doctor

**Published:** 1913-08-23

**Authors:** 


### EVERY MAN HIS OWN DOCTOR.

At the recent International Congress of Medicine Dr. A. L. Soresi, an American doctor, propounded the somewhat startling doctrine that a fair grounding in medical and surgical science should form part of every boy's (and girl's) education; and that thus, up to a point, everyone should be his own doctor. The fundamental argument on which this revolutionary idea is based is that preventive medicine nas progressed so far that, without the active co-operation of individual citizens, its further progress is likely to be slow. And such co-operation, Dr. Soresi concluded, is best elicited by educating the community wholesale in the outlines of medical and surgical science.

Both public and professional opinion are hardly ripe just yet for the acceptance of these views, we imagine, though every careful student of political and economic science must admit that they deserve consideration, and may be more feasible in ten or fifteen years' time. Meanwhile there is an increasing demand and sale for books which treat of matters medical in a popular way. More than one such volume has been reviewed in The Hospital within the last three or four years; and as a general rule we have satisfied ourselves that the task has been well done. The latest comer of such literature* is quite one of the best; it is by Dr. Woods Hutchinson, a relative (we believe) of the late Sir Jonathan Hutchinson, and a prominent figure at the recent Congress. Dr. Hutchinson practises in the United States, and his chapters are full of the quaint diction and striking catchwords so popular over there. There is, however, a fund of sound, common sense, clinical and practical wisdom, which the unaccustomed phrases merely serve to bring home more surely to the reader. We do not wish to convey the impression that there is anything in Dr. Woods Hutchinson's vocabulary approaching the strange jargon of the American newspaper reporter; his language is thoroughly clear and lucid. Moreover, he is obviously a man of wide and cultivated reading, and thoroughly well versed both in medicine and in human nature. Those who read through his always interesting pages will assuredly profit by them, for they will have a much clearer grasp of the problems which doctors have to solve and of their methods of grappling with the task. They will also get an acquaintance with the shifting sands of quackery which should deter them from venturing into that treacherous lure. In a word, they will get as near to fulfilling Dr. Soresi's ideal as it is expedient and profitable for them at the present time; for they can trust their guide, than which we can pay Dr. Hutchinson no higher compliment.

* *Common Diseases.* By Woods Hutchinson, M.A., M.D. Cassell and Co. 1913. Pp. 452. Price 6s. net.

